# Fungicide Dissipation Kinetics and Dietary Exposure Risk Assessment in Squash Fruit and Leaf

**DOI:** 10.3390/foods12061291

**Published:** 2023-03-17

**Authors:** Dai An, Rakdo Ko, Jinchan Kim, Kwanghun Lee, Ji-Ho Lee

**Affiliations:** 1 Korea Conformity Laboratories, Bio Division, Incheon 21999, Republic of Korea; 2 Department of Crop Sciences, Konkuk University, Seoul 05029, Republic of Korea

**Keywords:** *Cucurbita pepo*, squash leaf, dietary risk assessment, dissipation pattern, ultra-high-performance liquid chromatography–mass spectrometry, pesticide

## Abstract

The dissipation behavior and dietary exposure risk assessment of four fungicides (dimethomorph, mandipropamid, myclobutanil, and metalaxyl) was performed in fruits and leaves of squash grown under greenhouse conditions. Squash fruit and leaf samples were randomly collected at 0, 3, 5, 7, and 14 days after the last pesticide application. Analysis was performed using ultra-high performance liquid chromatography coupled with tandem mass spectrometry (UHPLC-MS/MS). The quick, easy, cheap, effective, rugged, and safe (QuEChERS) method was used for sample preparation. Recovery rates at two spiked levels (0.01 and 0.1 mg/kg) were found to be in the range of 76.4%–101.9% for the analyzed pesticides and their relative standard deviations were ≤4%. Pesticide half-lives were 2.1 and 4.9 days for dimethomorph, 4.6 and 8.1 days for mandipropamid, 4.7 and 8.2 days for myclobutanil, and 2.7 and 5 days for metalaxyl in squash fruit and leaf, respectively. Regarding the total surveyors, hazard quotient values for squash fruit and leaf were ≤1.03 × 10^−3^ and ≤2.39 × 10^−3^, respectively. These values in the case of true consumers were ≤3.14 × 10^−3^ and ≤3.91 × 10^−1^, respectively.

## 1. Introduction

Food safety is receiving more attention worldwide than ever before [1], and pesticide residues are a major harmful factor affecting human health [2]. Regarding human toxicity, an earlier study investigating the residual pesticides indicated that the exposure route is primarily through the ingestion of contaminated food and that pesticide intake through this route is considerably higher than that via any other route, such as air and water [3]. Although pesticides pose a potential risk to human health, they effectively protect crops against both pests and diseases, consequently increasing crop yield [4].

Pesticide residue levels in food are typically regulated to reduce unnecessary consumer exposure [5]. In the Republic of Korea (ROK), a positive list system (PLS) has been enforced since 2019 [6]. PLS represents a legislative rule that states that the tolerance limit of a pesticide residue (0.01 mg/kg) must be applied to agricultural products that have no maximum residue limit (MRL). Consumers can intake agricultural products that are safe [7]. While this system is a strict standard for producers, establishing the MRL is significant for both producers and consumers. Because major crops have a high economic value, pesticide manufacturers prioritize setting MRLs for major crops over minor crops [8].

Squash, a minor crop in the Cucurbitaceae family, is primarily consumed as fruit, leaf, and seed in the ROK. In the Fifth Korea National Health and Nutrition Examination Survey (5th KNHNES) [9], squash fruit was reported to be a major source of potassium and thiamine intake. Squash leaf is very often consumed in the ROK, and although the frequency of consumption is lower than that of squash fruit, it is known that its calcium content is approximately 6–45 times higher [10].

Dimethomorph and mandipropamid are carboxylic acid amide fungicides. According to microscopic and ultrastructural studies on mycelial cell endometrial structure, diemethomorph may disrupt the cell wall formation of pathogens [11]. Myclobutanil is a triazole fungicide that can interfere with the normal functions of male hormones and the CYP3A4 enzyme [12]. Metalaxyl has been found to stimulate the skin, accumulate in the liver, fat, and muscles, and induce thyroid tumors [13]. In the ROK, MRLs are currently established for several pesticides used for squash fruit and leaf [14]. However, in the European Union (EU), they are only established for squash fruit pesticides [15]. The MRL values for squash fruit in the EU and ROK are 0.5 and 1.5 mg/kg for dimethomorph, 0.3 mg/kg and undetermined for mandipropamid, 0.3 and 0.1 mg/kg for myclobutanil, and 0.01 and 0.2 mg/kg for metalaxyl, respectively. Previous studies have reported the dissipation pattern and exposure risk of chlorfenapyr [16], sulfoxaflor [17], tebuconazole, penconazole, myclobutnil, and triadimenol in squash fruit [18]. However, to the best of our knowledge, no studies to date have assessed the dissipation pattern or human exposure risk of any pesticide in squash leaf.

This study aimed to (1) develop and validate an ultra-high-performance liquid chromatography–mass spectrometry (UHPLC-MS/MS) method using a quick, easy, cheap, effective, rugged, and safe (QuEChERS) sample preparation procedure to determine the residue levels of four pesticides—dimethomorph, mandipropamid, myclobutanil, and metalaxyl—in squash fruit and leaf (2), calculate the half-life of the four pesticides based on their dissipation patterns, and (3) estimate the health risk to a true consumer by assessing pesticide residues. Experiments were conducted under greenhouse conditions, following which the four pesticides present in harvested squash fruits and leaves were analyzed. The results will provide a reference towards proposing usage recommendations for the four pesticides in squash fruit and leaf.

## 2. Materials and Methods

### 2.1. Chemicals, Reagents, and Materials

Dimethomorph, mandipropamid, myclobutanil, and metalaxyl were purchased from Kemidas Co. (Suwon, Republic of Korea). The chemical structures and physicochemical properties of the four pesticides are presented in Supplementary Material Table S1. Formulations of dimethomorph (wettable powder (WP), 25%, Dongbang Agro Corporation, Seoul, Republic of Korea), mandipropamid (suspension concentrate, 22.59%, Syngenta Korea Ltd., Jeonbuk, Republic of Korea), myclobutanil (WP, 6%, Kyung Nong Corporation, Seoul, Republic of Korea), and metalaxyl (WP, 25%, Enbio Co., Ltd., Gunpo-si, Republic of Korea) used in greenhouse experiments were purchased from a local market (Seoul, the ROK). HPLC-grade acetonitrile was provided by J. T. Baker (Phillipsburg, NJ, USA). Formic acid (>98% purity) was obtained from Merck KGaA (Darmstadt, Germany). Membrane filters (0.2 µm) were purchased from Phenomenex (Torrance, CA, USA). An EN-QuEChERS kit was purchased from Chiral Technology Korea (Daejeon, Republic of Korea).

### 2.2. Greenhouse Experiments

Squash plants were grown under greenhouse conditions. The greenhouse facility was located in Cheongju-si, Chungcheongbuk-do, the ROK (36.675120° N, 127.317190° E). The experiments were conducted in accordance with the relevant institutional, national, and international Good Laboratory Practice guidelines and legislation [19]. Each pesticide treatment plot measured 45 m (length) × 1 m (width), divided into three replicates. A control plot was located in an area separated from the treated areas. Each experimental plot was treated with one of the four pesticides three times before harvest. The applications were performed per 7 days, and the first application was performed on 6 April 2021. According to the safe use standard of agricultural chemicals, dimethomorph, mandipropamid, myclobutanil, and metalaxyl formulations were diluted in water in ratios of 1:1000, 1:2000, 1:1000, and 1:1500, respectively, and were applied on squash fruit and leaf by spraying at recommended dosages of 45, 20.331, 7.2, and 45 g a.i./10a, respectively [10]. The total spraying volume was 27.5 L per plot (45 m^2^). Squash fruit samples (>2 kg) and squash leaf samples (>1 kg) were collected 0, 3, 5, 7, and 14 days (including the pre-harvest interval (PHI)) after the last pesticide application, randomly from each replicate. During cultivation, the greenhouse air temperature range and humidity were maintained at 13.7 °C–20.9 °C and 63.0%–90.9%, respectively (Supplementary Material Table S2), measured using an electric data logger (TandD, T&D, Matsumoto, Japan). After harvesting, all plant samples were immediately transferred to the laboratory. Squash fruit and leaf samples were homogenized in dry ice and subsequently stored at −20 °C in polyethylene bags.

### 2.3. Standard Solutions

Standard stock solutions of the four pesticides were prepared at a concentration of 1000 mg/L in acetonitrile. Working solutions at concentrations of 0.01, 0.02, 0.04, 0.1, and 0.2 mg/L were prepared by the serial dilution of the stock solutions with acetonitrile. Each of the working solutions was mixed with the extract of squash fruit or squash leaf in a ratio of 1:1 to prepare the matrix-matched standard solutions of 0.005, 0.01, 0.02, 0.05, and 0.1 mg/L.

### 2.4. Sample Preparation and Extraction

An EN-QuEChERS kit [20] was used to extract the pesticides. Homogenized squash fruits or squash leaves (10 g) were weighed in 50 mL falcon tubes. Ten milliliters of acetonitrile were added to each tube. The EN-QuEChERS kit reagent (4 g MgSO_4_, 1 g NaCl, 1 g sodium citrate, and 0.5 g disodium citrate sesquihydrate) was added into the falcon tubes. The tubes were shaken at 1200 rpm for 1 min. After centrifugation at 4000 rpm for 5 min, 1 mL of the supernatant was filtered using a 0.2 μm PTFE syringe filter.

### 2.5. LC-MS/MS Analytical Conditions

All samples were analyzed using the Shimadzu LCMS-8045 device equipped with a UHPLC Nexera X2 system (Kyoto, Japan) coupled with a Kinetex C18 column (2.1 × 150 mm; 2.6 μm particle size; Phenomenex, California, USA). The column oven temperature was maintained at 40 °C and the injection volume was 5 μL (for metalaxyl) or 2 μL (for other pesticides). Mobile phases consisted of water with 0.1% formic acid (A) and acetonitrile with 0.1% formic acid (B). The total mobile phase flow rate was 0.2 mL/min. The following gradient conditions were used (A:B): for dimethomorph—90:10 (0–0.5 min), 55:45 (1–5.5 min), 10:90 (6–6.5 min), and 90:10 (7–8 min); for other pesticides—90:10 (0–1 min), 90:10 (2–5 min), and 90:10 (6–7 min).

The following MS instrumental conditions with a positive ESI source were applied: an interface temperature of 350 °C, a heat block temperature of 400 °C, a nebulizing gas flow rate of 3 L/min, and a drying gas flow rate of 10 L/min. The four pesticides were analyzed in the multiple reaction monitoring mode using a triple quadrupole (QqQ) mass spectrometer (Shimadzu, Japan) (Table 1).

### 2.6. Method Validation

The method was validated in terms of the linearity of calibration curves and the recovery test through limits of quantitation (LOQs). Matrix-matched calibration was examined using prepared matrix-matched standard solutions of 0.005, 0.01, 0.02, 0.05, and 0.1 mg/L. Among the chromatograms that produced a signal-to-noise ratio (S/N) of >10, the lowest concentration was defined as the LOQ that could enable the quantitation of the target compound. To estimate the accuracy and precision through relative standard deviation (RSD) values, the recovery test was performed at two different concentrations: 0.01 and 0.1 mg/kg. Each test was performed in triplicate. Only the parent substance was evaluated, except for dimethomorph residues. The level of dimethomorph residue in the plants was calculated as the sum of (E)-dimethomorph and (Z)-dimethomorph, which are isomers of dimethomorph.

### 2.7. Statistical Analysis

The dissipation patterns of the four pesticides in the squash fruit and leaf over time were expressed by the following first-order kinetics (Equation (1)), and the half-lives were calculated using Equation (2) [21]:

(1) $$C_{t}=C_{0}\times e^{-kt}$$

(2) $$t_{1/2}=\ln\times 2/k,$$

where *C*_0_ is the initial residue concentration of a pesticide (mg/kg), which represents the residue levels 2 h after the last treatment, *C_t_* is the residue concentration (mg/kg), *t* represents days after the last spraying, and *k* is the constant rate of dissipation.

### 2.8. Dietary Risk Assessment

The human health risk related to pesticide residues present in squash fruit and leaf was estimated though the dietary intake rate compared to acceptable daily intake (ADI) [22,23]. Hazard quotient (HQ) based on estimated daily intake (EDI) and ADI was calculated following Equations (3) and (4):

(3) $$EDI=C_{t}\times IR/BW$$

(4) HQ=EDI/ADI,

where EDI represents the estimated daily intake (mg/kg b.w./day), *C_t_* is the residue concentration of a pesticide in squash fruit or leaf (mg/kg), and IR is the average squash fruit or leaf intake (kg/day) that was obtained from the Standardization Guidelines for Food Intake Calculation and Contamination Monitoring conducted in 2019 by the National Institute of Food and Drug Safety Evaluation on 43,602 individuals. The average consumption of squash fruit and leaf was 0.01042 kg and 0.0018 kg per day per person, respectively [24]. Among the total surveyors (n = 43,602), the true consumers of squash fruit were 14,013 individuals (32.14%). The average squash fruit intake of a true consumer (n = 14,013) was 0.03188 kg per day. With regard to squash leaf, the true consumer percentage among the total surveyors was 0.73% and the average squash leaf intake of a true consumer (n = 319) was 0.02946 kg per day. The body weight was obtained from 5^th^KNHNES [12], and the average weight of total surveyors was 59.44 kg. The ADI values of dimethomorph, mandipropamid, myclobutanil, and metalaxyl are 0.2, 0.05, 0.03, and 0.08 mg/kg b.w./day, respectively [14]. If a value of <1 indicates that a population is at no risk of exposure. All statistical data for the study were obtained from a publicly accessible database.

## 3. Results

### 3.1. Method Validation

**Figure 1:**
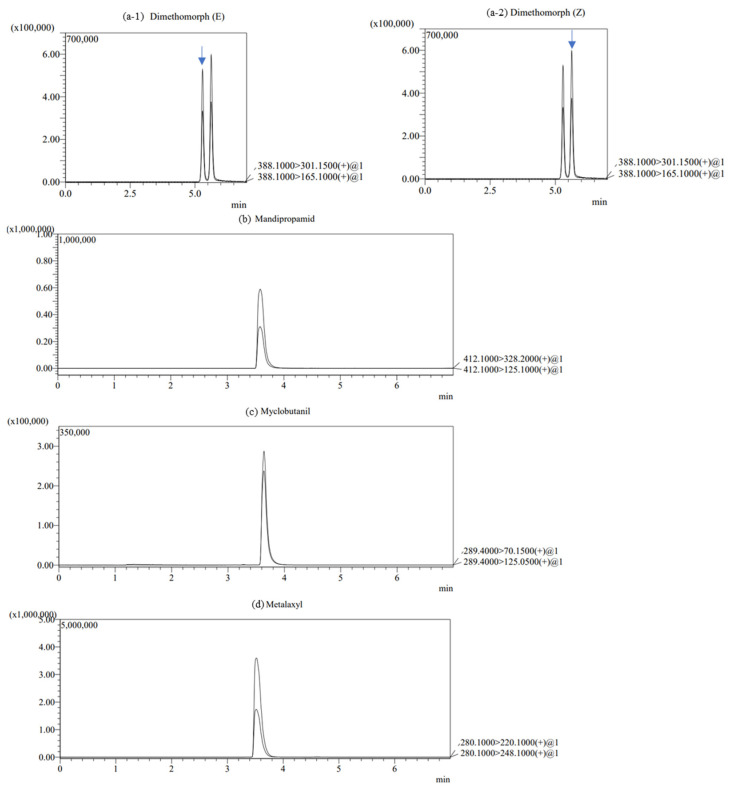
Representative chromatogram (0.1 mg/kg) of (**a-1**) dimethomorph (E), (**a-2**) dimethomorph (Z), (**b**) mandipropamid, (**c**) myclobutanil, (**d**) metalaxyl.

Method validation was performed in terms of linearity, accuracy, and precision. The calibration curves of all pesticides were linear from 0.005 to 0.1 mg/L of standard solutions in both squash fruit and leaf (Table 2, Figure 1). MLOQ was 0.01 mg/kg for all pesticides. The accuracy and precision were determined using the recovery rate and RSD of recovery tests performed in triplicate at two different concentrations (0.01 and 0.1 mg/kg). The results presented in Table 3 show satisfactory recovery rates and RSD for two spiked levels in squash fruit and leaf for all pesticides. The range of recovery rates was 76.4%–101.9% at two different concentrations. The RSD values for all pesticides were less than 4% (Table 3).

### 3.2. Pesticides MRLs in the EU and ROK

#### 3.2.1. MRL in Squash Fruit

MRL values in the EU and ROK for squash fruit are presented in Table 4. Table 4 also shows the pesticide residue levels at 0, 3, 5, 7, and 14 days after the last application. The residue levels of the dimethomorph on days 0 and 7 of application were 0.36 and 0.02 mg/kg, respectively, which are 24% and 1.3% of the national MRL (1.5 mg/kg) and 72% and 40% of the MRL set in the EU (0.5 mg/kg). The residue levels of mandipropamid on days 0 and 7 of application were 0.29 and 0.08 mg/kg, respectively, which are 97% and 27% of the MRL set in the EU (0.3 mg/kg). Regarding myclobutanil, the residue levels on days 0 and 7 of application were 0.06 and 0.01 mg/kg, respectively, which are 60% and 10% of the national MRL (0.1 mg/kg), and 20% and 33% of the MRL set in the EU (0.3 mg/kg). The residue levels of metalaxyl on days 0 and 7 of application were 0.33 and 0.05 mg/kg, respectively, which are 165% and 25% of the national MRL (0.2 mg/kg) and 3300% and 500% of the MRL set in the EU (0.01 mg/kg). In the ROK, the MRL of mandipropamid in squash has not yet been established. Hence, our residual level data for mandipropamid may be used as a basis for the establishment of a national MRL. Dimethomorph, myclobutanil, and metalaxyl MRLs in squash fruit differ between the ROK and EU. Pesticide residual levels can differ depending on plant growth conditions (air temperature and humidity), climate, and crop species [25,26,27].

#### 3.2.2. MRL in Squash Leaf

Currently, in the ROK, MRL is only established for myclobutanil and metalaxyl for squash leaf [14], and in the EU, none of the pesticides have a defined MRL for squash leaf [15]. Therefore, the initial residue levels (mg/kg) of myclobutanil and metalaxyl were compared to the national MRLs. The initial residue level of myclobutanil was 11.65 mg/kg, which is 58% of the national MRL (20 mg/kg), and that of metalaxyl was 31.71 mg/kg, which is 106% of the national MRL (30 mg/kg). Regarding dimethomorph and mandipropamid, the initial residue levels were 69.88 and 39.41 mg/kg, respectively. While the residue levels of myclobutanil and metalaxyl on day 7 of the application were 6.07 (30%) and 8.09 mg/kg (27%), respectively, which are less than 20 and 30 mg/kg (the national MRL for each pesticide, respectively), the residue levels of the dimethomorph and mandipropamid on day 7 of application were 25.97 and 16.86 mg/kg, respectively. The obtained residual level data for these pesticides in squash leaf may be used as a basis for the establishment of their MRLs.

### 3.3. Residual Characteristics of Pesticides in Squash Fruit and Leaf

**Figure 2:**
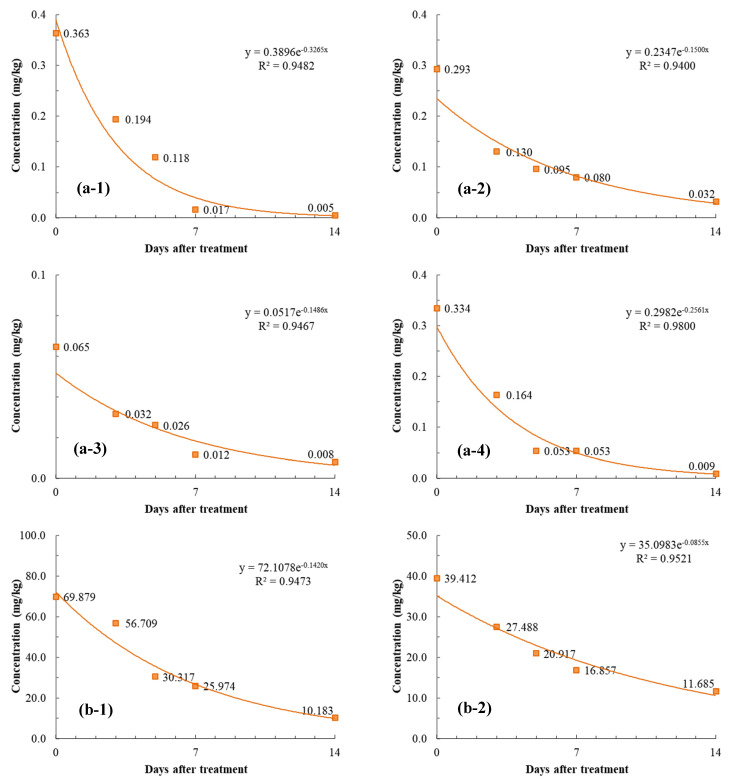

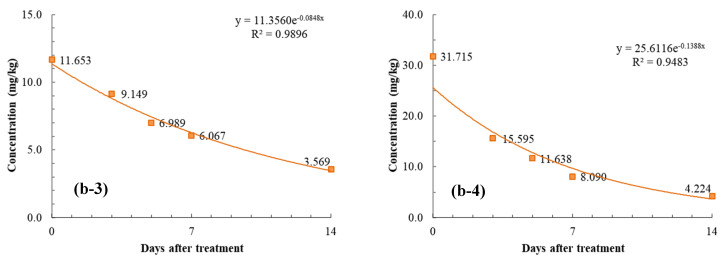
In squash fruit, dissipation patterns of (**a-1**) dimethomorph, (**a-2**) mandipropamid, (**a-3**) myclobutanil, (**a-4**) metalaxyl. In squash leaf, dissipation patterns of (**b-1**) dimethomorph, (**b-2**) mandipropamid, (**b-3**) myclobutanil, (**b-4**) metalaxyl.

The residue dissipation patterns of the four pesticides applied in a greenhouse are shown in Figure 2. The data imply that the pesticide residue levels were closely related to the specific surface area and texture of the crop. Because squash fruit has a smaller specific surface area and a smoother texture than squash leaf, they show a large difference in initial residue levels, despite being sprayed under the same cultivation conditions. Specifically, the initial residue levels in squash fruit 2 h after the last treatment were 0.36, 0.29, 0.06, and 0.33 mg/kg for dimethomorph, mandipropamid, myclobutanil, and metalaxyl, respectively, while those in squash leaf were 69.88, 39.41, 11.65, and 31.71 mg/kg, respectively. By comparing the initial residual levels between the two organs, these values in squash leaf were found to be 193, 135, 180, and 95 times higher for dimethomorph, mandipropamid, myclobutanil, and metalaxyl, respectively.

The residual levels of all pesticides decreased as the harvest date after the last spraying was postponed, and the correlation coefficients of dissipation kinetics were in the range of 0.94–0.98 (Figure 2). Figure 2 show the regression curves and dissipation equations of the four pesticides evaluated by first-order kinetic analysis in squash fruit and leaf. The half-lives of all pesticides were shorter in fruit than in leaf. In this study, it is hypothesized that the significant difference observed between the pesticides’ half-life in squash fruit and leaf could be due to the effect of dilution during plant growth [28].

In squash fruit, the half-lives of dimethomorph, mandipropamid, myclobutanil, and metalaxyl were 2.1, 4.6, 4.7, and 2.7 days, respectively. In previous studies, the half-lives of various pesticides were reported: chlorfenapyr (3.05 days) [16], sulfoxaflor (6.13 days) [17], tebuconazole (2.30 days), triadimenol (2.81 days), and myclobutanil (2.98 days) [18]. In field experiments, increasing temperature accelerates numerous processes associated with pesticide dissipation [28,29,30,31]. Moreover, the higher the air humidity, the higher the pesticide adsorption affinity to the plant surface and the lower its volatilization [32,33]. In a previous study on myclobutanil residues in squash fruit [18], the average temperature and average humidity during field experiments were reported to be 13 °C and 60%, respectively. In this study, the average temperature of 18 °C and the average humidity of 71% were relatively high and likely contributed to the short half-life of the pesticide; however, various factors involved in its half-life (e.g., microbial activity, rainfall, and sunlight) appear to have been combined. The half-lives of dimethomorph, mandipropamid, myclobutanil, and metalaxyl in squash leaf were 4.9, 8.1, 8.2, and 5 days, respectively. Consequently, their half-lives were higher in squash leaf than in squash fruit. Unlike squash fruit, no studies have been reported on the half-lives of these pesticides in squash leaf.

Previous studies have shown that dimethomorph tends to decompose quickly in iceberg lettuce (0.86 days) [34] and pepper (3.2–3.8 days) [35], but slowly in potato (9.4 days) [36], grape (9–9.8 days) [37], and pak choi (6.2 days) [38]. Mandipropamid has been reported to have a short or a similar half-life in grape (2.2 days) [39], Korean cabbage (3.9–4 days) [40], and sesame leaf (5.1–5.4 days) [41]. A few studies on myclobutanil half-life showed similar trends, reporting 2.2–3.4 days for lychee [42], 2.5–4.5 days for wheat [43], and 4.9–6.8 days for green tobacco leaf [44]. Regarding the chemical classes, the dissipation half-lives of triazoles were reported to be in the range of 2–12.8 days [45], which is similar to our results and previous studies. The half-life of metalaxyl was reported to be 2.5 days in Chinese cabbage [46], 3–3.5 days in cucumber [47], 4.9 days in grape [37], and 16.5 days in durian leaf [48]. The decomposition behavior of pesticides is generally influenced by many factors such as their physicochemical characteristics, formulations, environmental conditions, crop species, and plant growth-based dilution factor [29,49,50].

### 3.4. Dietary Risk Assessment

The dietary risk assessment was performed by calculating the dietary intake rates compared to ADI. An HQ value of >1 indicates that a population is at risk of exposure. Table 5 shows the risk assessment results of four fungicides on days 0, 3, and 7 after the last treatment for total surveyors and true consumers associated with squash fruit and leaf consumption. Considering the total surveyors (n = 43,602), the HQ values of squash fruit on days 0, 3, and 7 of application were ≤1.03 × 10^−e^ and those of squash leaf on days 0, 3, and 7 of the application were ≤2.39 × 10^−3^ (Table 5a). Because there was a large difference in squash intake between the actual consumers and non-consumers, additional risk assessments were performed in true consumers. The resulting HQ values of squash fruit on days 0, 3, and 7 of application were ≤3.14 × 10^−3^ and those of squash leaf on days 0, 3, and 7 of the application were ≤3.91 × 10^−e^ (Table 5b). The PHI was 3, 7, and 7 days for dimethomorph, myclobutanil, and metalaxyl, respectively. During the PHI period, the HQ values of all three pesticides were <l. The PHI of mandipropamid in squash fruit and leaf has not been established in the ROK. The mandipropamid risk assessment results can help contribute toward the establishment of its PHI. Our findings imply that the exposure risk of true consumers of squash fruit and leaf to these pesticides was 3 times and 164 times higher, respectively, than that of all surveyors. According to the national nutrition statistics of the Korea Health Industry Development Institute [51], the intake of squash fruit and squash leaf increases after the age of 30, and it was found that the population of small/medium-sized cities and suburbs consumed 6 and 16.5 times more squash leaf than the population of large cities, respectively. When the average intake was examined according to the age and settlement type, it was found to be higher for individuals residing in the suburbs than for those residing in large cities as well as for older individuals. Considering the daily consumption of squash fruit and leaf, the exposure risk to pesticides is regarded as safe.

## 4. Conclusions

We studied the residual levels and dissipation patterns and performed the dietary risk assessment of four fungicides—dimethomorph, mandipropamid, myclobutanil, and metalaxyl—in squash fruit and leaf during greenhouse cultivation. To assess the pesticide residues, simple and rapid analytical methods based on a modified QuEChERS method were developed and validated using UHPLC-MS/MS. Based on the obtained HQ values, it can be concluded that the intake of pesticide residues from squash fruit and leaf does not pose a health risk to even a true consumer. Overall, it is expected that these results will help us understand the properties of dimethomorph, mandipropamid, myclobutanil, and metalaxyl residues as well as establish their domestic MRLs.

## Figures and Tables

**Table 1:** Multiple reaction monitoring conditions of the four analytes.

Analyte	Ionization	Precursor Ion (*m/z*)	Product Ion (*m/z*)		Retention Time (min)
			Quantitation (Collision Energy, eV)	Qualification (Collision Energy, eV)	
Dimethomorph	[M + H]^+^	388.1	301.15 (21)	165.1 (33)	5.3; (E)-isomer
					5.6; (Z)-isomer
Mandipropamid	[M + H]^+^	412.1	328.2 (15)	125.1 (35)	3.5
Myclobutanil	[M + H]^+^	289.4	70.15 (18)	125.05 (31)	3.6
Metalaxyl	[M + H]^+^	280.1	220.1 (14)	248.1 (11)	3.5

**Table 2:** Linear equation of the calibration curves for the quantification of the four pesticide residues in squash fruit and leaf.

Pesticide	Linear Range (mg/L)	Squash Fruit		Squash Leaf	
		Linear Equation	R^2^	Linear Equation	R^2^
Dimethomorph	0.005–0.1	y = 31,386,895.5097 × 8396.1323	0.9993	y = 27,666,075.9306 × 34,369.7811	0.9996
Mandipropamid		y = 23,414,787.2168 × 8024.7460	0.9951	y = 15,504,048.6650 × 9435.4988	0.9997
Myclobutanil		y = 8,773,295.1861 × 9848.4438	0.9965	y = 7,088,559.9919 × 3798.8394	0.9923
Metalaxyl		y = 145,612,794.4175 × 276,181.3131	0.9987	y = 61,474,433.9401 × 146,444.2884	0.9992

**Table 3:** Recovery tests in squash fruit and leaf.

Pesticide	Squash Fruit				Squash Leaf			
	Added Concentration (mg/kg)				Added Concentration (mg/kg)			
	0.01 mg/kg		0.1 mg/kg		0.01 mg/kg		0.1 mg/kg	
	Recovery Mean (%)	RSD	Recovery Mean (%)	RSD	Recovery Mean (%)	RSD	Recovery Mean (%)	RSD
Dimethomorph	98.0	1.1	100.0	1.7	92.3	1.0	98.8	0.3
Mandipropamid	85.7	3.5	95.4	0.8	87.9	3.0	89.3	3.6
Myclobutanil	99.9	1.5	96.7	1.7	88.5	4.5	89.8	1.7
Metalaxyl	87.6	1.2	96.5	0.7	76.6	0.3	94.0	1.4

RSD, relative standard deviation.

**Table 4:** (**a**) Average residues of four pesticides in squash fruit. (**b**) Average residues of four pesticides in squash leaf.

(a)				
Pesticide	Days after the Last Treatment	Mean ± SD (mg/kg)	EU MRL of Squash Fruit (mg/kg)	National MRL of Squash Fruit (mg/kg)
Dimethomorph	0	0.36 ± 0.00	0.5	1.5
	3	0.19 ± 0.01		
	5	0.12 ± 0.02		
	7	0.02 ± 0.00		
	14	0.00 ± 0.00		
Mandipropamid	0	0.29 ± 0.04	0.3	-
	3	0.13 ± 0.02		
	5	0.10 ± 0.00		
	7	0.08 ± 0.00		
	14	0.03 ± 0.00		
Myclobutanil	0	0.06 ± 0.00	0.3	0.1
	3	0.03 ± 0.00		
	5	0.03 ± 0.00		
	7	0.01 ± 0.00		
	14	0.01 ± 0.00		
Metalaxyl	0	0.33 ± 0.02	0.01	0.2
	3	0.16 ± 0.03		
	5	0.05 ± 0.01		
	7	0.05 ± 0.01		
	14	0.01 ± 0.00		
**(b)**				
**Pesticide**	**Days after the Last Treatment**	**Mean ± SD (mg/kg)**	**EU MRL of Squash Leaf (mg/kg)**	**National MRL of Squash Leaf (mg/kg)**
Dimethomorph	0	69.88 ± 6.46	-	-
	3	56.71 ± 3.06		
	5	30.32 ± 1.57		
	7	25.97 ± 1.55		
	14	10.18 ± 3.23		
Mandipropamid	0	39.41 ± 4.22	-	-
	3	27.49 ± 0.85		
	5	20.92 ± 4.09		
	7	16.86 ± 1.53		
	14	11.69 ± 1.60		
Myclobutanil	0	11.65 ± 1.52	-	20
	3	9.15 ± 0.65		
	5	6.99 ± 0.38		
	7	6.07 ± 0.93		
	14	3.57 ± 0.32		
Metalaxyl	0	31.71 ± 0.54	-	30
	3	15.59 ± 0.77		
	5	11.64 ± 1.03		
	7	8.09 ± 1.38		
	14	4.22 ± 0.23		

SD, standard deviation; EU, European Union; MRL, maximum residue limit.

**Table 5:** (**a**) HQ of four pesticides in total surveyors related to squash fruit and leaf consumption. (**b**) HQ of four pesticides in true consumers related to squash fruit and leaf consumption.

(a)						
Pesticide			Residue Value (mg/kg)	ADI	EDI (mg/kg b.w./day)	HQ
Dimethomorph	Day 0	Fruit	0.36	0.2	6.31 × 10^−5^	3.16 × 10^−4^
		Leaf	69.88		2.12 × 10^−4^	1.06 × 10^−3^
	Day 3	Fruit	0.19		3.33 × 10^−5^	1.67 × 10^−4^
		Leaf	56.71		1.72 × 10^−4^	8.59 × 10^−4^
	Day 7	Fruit	0.02		3.51 × 10^−6^	1.75 × 10^−5^
		Leaf	25.97		7.86 × 10^−5^	3.93 × 10^−4^
Mandipropamid	Day 0	Fruit	0.29	0.05	5.08 × 10^−5^	1.02 × 10^−3^
		Leaf	39.41		1.19 × 10^−4^	2.39 × 10^−3^
	Day 3	Fruit	0.13		2.28 × 10^−5^	4.56 × 10^−4^
		Leaf	27.49		8.32 × 10^−5^	1.66 × 10^−3^
	Day 7	Fruit	0.08		1.40 × 10^−5^	2.80 × 10^−4^
		Leaf	16.86		5.11 × 10^−5^	1.02 × 10^−3^
Myclobutanil	Day 0	Fruit	0.06	0.04	1.05 × 10^−5^	2.63 × 10^−4^
		Leaf	11.65		3.53 × 10^−5^	8.82 × 10^−4^
	Day 3	Fruit	0.03		5.26 × 10^−6^	1.31 × 10^−4^
		Leaf	9.15		2.77 × 10^−5^	6.93 × 10^−4^
	Day 7	Fruit	0.01		1.75 × 10^−6^	4.38 × 10^−5^
		Leaf	6.07		1.84 × 10^−5^	4.60 × 10^−4^
Metalaxyl	Day 0	Fruit	0.33	0.07	5.78 × 10^−5^	8.26 × 10^−4^
		Leaf	31.71		9.60 × 10^−5^	1.37 × 10^−3^
	Day 3	Fruit	0.16		2.80 × 10^−5^	4.01 × 10^−4^
		Leaf	15.59		4.72 × 10^−5^	6.74 × 10^−4^
	Day 7	Fruit	0.05		8.77 × 10^−6^	1.25 × 10^−4^
		Leaf	8.09		2.45 × 10^−5^	3.50 × 10^−4^
**(b)**						
**Pesticide**			**Residue Value (mg/kg)**	**ADI**	**EDI (mg/kg b.w./day)**	**HQ**
Dimethomorph	Day 0	Fruit	0.36	0.2	1.93 × 10^−4^	9.65 × 10^−4^
		Leaf	69.88		3.46 × 10^−2^	1.73 × 10^−1^
	Day 3	Fruit	0.19		1.02 × 10^−4^	5.10 × 10^−4^
		Leaf	56.71		2.81 × 10^−2^	1.41 × 10^−1^
	Day 7	Fruit	0.02		1.07 × 10^−5^	5.36 × 10^−5^
		Leaf	25.97		1.29 × 10^−2^	6.44 × 10^−2^
Mandipropamid	Day 0	Fruit	0.29	0.05	1.56 × 10^−4^	3.11 × 10^−3^
		Leaf	39.41		1.95 × 10^−2^	3.91 × 10^−1^
	Day 3	Fruit	0.13		6.97 × 10^−5^	1.39 × 10^−3^
		Leaf	27.49		1.36 × 10^−2^	2.72 × 10^−1^
	Day 7	Fruit	0.08		4.29 × 10^−5^	8.58 × 10^−4^
		Leaf	16.86		8.36 × 10^−3^	1.67 × 10^−1^
Myclobutanil	Day 0	Fruit	0.06	0.04	3.22 × 10^−5^	8.05 × 10^−4^
		Leaf	11.65		5.77 × 10^−3^	1.44 × 10^−1^
	Day 3	Fruit	0.03		1.61 × 10^−5^	4.02 × 10^−4^
		Leaf	9.15		4.53 × 10^−3^	1.13 × 10^−1^
	Day 7	Fruit	0.01		5.36 × 10^−6^	1.34 × 10^−4^
		Leaf	6.07		3.01 × 10^−3^	7.52 × 10^−2^
Metalaxyl	Day 0	Fruit	0.33	0.07	1.77 × 10^−4^	2.53 × 10^−3^
		Leaf	31.71		1.57 × 10^−2^	2.25 × 10^−1^
	Day 3	Fruit	0.16		8.58 × 10^−5^	1.23 × 10^−3^
		Leaf	15.59		7.73 × 10^−3^	1.10 × 10^−1^
	Day 7	Fruit	0.05		2.68 × 10^−5^	3.83 × 10^−4^
		Leaf	8.09		4.01 × 10^−3^	5.73 × 10^−2^

ADI, acceptable daily intake; EDI, estimated daily intake; HQ, hazard quotient.

## Data Availability

Data will be made available on request.

## Supplementary Materials

The following supporting information can be downloaded at: https://www.mdpi.com/article/10.3390/foods12061291/s1, Table S1: Chemical structures and physicochemical properties of the four pesticides.; Table S2: Greenhouse air temperature, humidity, and pesticide treatment date during cultivation of squash plants.

## Abbreviations

ADI	Acceptable daily intake
CV	Coefficients of variation
EDI	Estimated daily intake
HPLC	High-performance liquid chromatography
HQ	Hazard quotient
LC-MS/MS	Liquid chromatography coupled with tandem mass spectrometry
LOQ	Limit of quantitation
MLOQ	Method limit of quantification
MRL	Maximum residue limit
PHI	Pre-harvest interval
