# Supplementary material for: Fungicide Dissipation Kinetics and Dietary Exposure Risk Assessment in Squash Fruit and Leaf
Source: Foods. 2023 Mar 17;12(6):1291. doi: 10.3390/foods12061291 (PMC10048305; doi:10.3390/foods12061291)
Supplement: Supplementary file 1 [file foods-12-01291-s001.zip › foods-2240577-supplementary.pdf]

Table S1. Chemical structures and physicochemical properties of the four pesticides.

**Pesticide**	**Chemical structure**	**Molecular weight**	**Vapor pressure (mPa)**
Dimethomorph	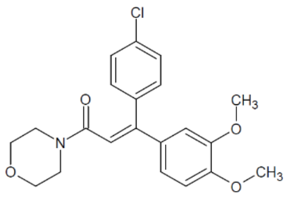	387.9	9.7×10^{-4}; (E)-isomer<br>1.0×10^{-3}; (Z)-isomer<br>(25 °C)
Mandipropamid	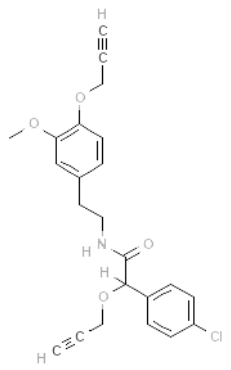	411.9	9.4×10^{-4} (25°C)
Myclobutanil	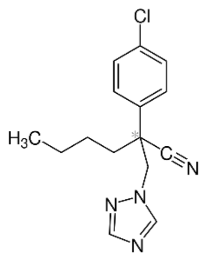	288.8	1.98×10^{-1} (20°C)
Metalaxyl	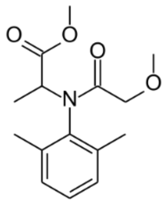	279.3	0.75 (25°C)

Table S2. Greenhouse air temperature, humidity, and pesticide treatment date during cultivation of squash plants.

Date	Temperature (°C)			Average humidity (%)	Pesticide treatment and sampling date
	Minimum	Maximum	Average		
April 6, 2021	4.3	35.2	17.0	68.6	First treatment
April 7, 2021	3.9	36.2	17.6	63.0	
April 8, 2021	3.5	33.6	16.7	63.8	
April 9, 2021	2.8	37.5	15.9	65.4	
April 10, 2021	3.8	35.3	17.1	67.1	
April 11, 2021	5.0	36.5	19.0	68.4	
April 12, 2021	11.5	26.8	15.7	90.9	
April 13, 2021	7.9	40.0	20.1	81.2	Second treatment
April 14, 2021	4.1	31.4	16.1	66.0	
April 15, 2021	2.1	32.8	15.5	67.3	
April 16, 2021	6.0	27.8	13.7	88.8	
April 17, 2021	4.8	36.7	14.1	73.3	
April 18, 2021	4.7	31.2	15.6	70.2	
April 19, 2021	2.2	34.7	16.0	63.8	
April 20, 2021	3.8	33.8	18.1	66.0	Third treatment and 0 day sampling after 2 h
April 21, 2021	6.5	41.0	22.1	65.6	
April 22, 2021	14.1	38.0	22.4	63.5	
April 23, 2021	14.3	27.7	19.1	53.3	3 days sampling
April 24, 2021	10.2	36.2	20.0	69.8	
April 25, 2021	7.7	38.0	20.9	67.5	5 days sampling
April 26, 2021	6.5	33.3	19.3	64.0	
April 27, 2021	11.3	29.1	18.5	77.2	7 days sampling
April 28, 2021	11.0	27.4	18.4	76.7	
April 29, 2021	9.5	29.6	18.2	75.1	
April 30, 2021	10.7	29.8	17.5	82.6	
May 1, 2021	10.1	36.6	15.8	88.9	
May 2, 2021	7.2	32.2	17.7	71.8	

May 3, 2021	4.1	32.9	18.5	69.0	
May 4, 2021	11.2	25.8	18.2	75.2	14 days sampling
